# γδ T Lymphocytes: An Effector Cell in Autoimmunity and Infection

**DOI:** 10.3389/fimmu.2018.02389

**Published:** 2018-10-16

**Authors:** Carolina Maiumi Shiromizu, Carolina Cristina Jancic

**Affiliations:** ^1^Laboratorio de Inmunidad Innata, Instituto de Medicina Experimental (IMEX) — CONICET, Academia Nacional de Medicina, Buenos Aires, Argentina; ^2^Departamento de Microbiología, Parasitología e Inmunología, Facultad de Medicina, Universidad de Buenos Aires, Buenos Aires, Argentina

**Keywords:** γδ T lymphocytes, inflammation, autoimmunity, infection, innate cells

## Abstract

γδ T cells are non-conventional lymphocytes which show several properties of innate immune cells. They present a limited TCR repertoire and circulate as cells with a pre-activated phenotype thus being able to generate rapid immune responses. γδ T cells do not recognize classical peptide antigens, their TCRs are non-MHC restricted and they can respond to pathogen-associated molecular patterns and to cytokines in absence of TCR ligands. They also recognize self-molecules induced by stress, which indicate infection and cellular transformation. All these features let γδ T cells act as a first line of defense in sterile and non-sterile inflammation. γδ T cells represent 1–10% of circulating lymphocytes in the adult human peripheral blood, they are widely localized in non-lymphoid tissues and constitute the majority of immune cells in some epithelial surfaces, where they participate in the maintenance of the epithelial barriers. γδ T cells produce a wide range of cytokines that orchestrate the course of immune responses and also exert high cytotoxic activity against infected and transformed cells. In contrast to their beneficial role during infection, γδ T cells are also implicated in the development and progression of autoimmune diseases. Interestingly, several functions of γδ T cells are susceptible to modulation by interaction with other cells. In this review, we give an overview of the γδ T cell participation in infection and autoimmunity. We also revise the underlying mechanisms that modulate γδ T cell function that might provide tools to control pathological immune responses.

## Introduction

γδ T cells are non-conventional T lymphocytes present in blood and tissues with a restricted TCR repertoire. During the ontogeny in the thymus, γδ T cells develop before αβ T lymphocytes and are abundant during the first weeks of fetal development. However, after birth, they constitute a minor fraction of thymocytes. This is similar in humans and rodents (1). In healthy adult humans, they represent 1–10% of the total circulating lymphocytes with a phenotype mainly CD4/CD8 double negative (2). They are found in high proportion in epithelial tissues, being particularly abundant in the intestine (3). In homeostatic conditions, γδ T cells can display a pre-activated and memory phenotype and the high frequency of these cells enables rapid responses without the presence of cognate TCR agonists and/or cellular expansion (1). γδ T cells can recognize many microorganisms and infected or transformed host cells (4) and exert a direct cytotoxic activity, which involves secretory, and non-secretory pathways, i.e., the release of granzymes and perforins and the engagement of Fas and TNF-related apoptosis-inducing ligand receptors, respectively (5–7). Moreover, γδ T cells can propitiate the healing of damaged tissues and maintain the epithelial integrity (8). They can also generate memory cells, hence acting like adaptive immune T cells (9). Interestingly, and similar to conventional T lymphocytes, γδ T cells can differentiate into different effector profiles, and produce different chemokines and a wide array of cytokines including IFN-γ, TNF-α, IL-17, IL-21, and IL-22 (10). Recently, it has been reported in a murine model that in adipose tissue γδ T cells are abundant and they participate in the regulation of body temperature, through the production of IL-17A and TNF-α, and through the maintenance of catecholamine sensitivity for lipolysis induction. Moreover, in adipose tissue, γδ T cells let the recruitment and homeostatic expansion of regulatory T cells (11).

Regarding the effector profiles in mice, γδ T cells complete their functional differentiation in the fetal thymus (12). It has been shown, that γδ T cells that bind antigens with low affinity will produce IL-17, while those that bind antigen with high affinity will secrete IFN-γ (13). Another difference between human and mouse γδ T cells is their classification. In humans, γδ T cells are classified according to their Vδ gene segment used. Until now only three true Vδ genes exist: Vδ1-3; and seven functional Vγ gene segments: Vγ2-5, Vγ8, Vγ9, and Vγ11. While in mice γδ T cell subsets are named according to the Vγ chain used (14). Of note, the data describing the γδ T cell subsets of a particular species cannot be translated directly to another species because each repertoire is unique. The γδ TCR repertoire is restricted and is associated with the tissue distribution (5). The limited γδ TCR repertoire is consistent with their capacity to recognize conserved pathogen-derived antigens and self-molecules expressed under cellular stress conditions (5). Their tissue distribution and their capacity to recognize and rapidly respond to self- and non-self-conserved antigens allow them to act as the first line of defense in peripheral tissues (4). In humans, Vδ1+ T cells are abundant in the epithelium (8, 15, 16), they recognize molecules of the non-classical MHC family, either with or without loaded antigens, such as CD1a, c and d; and the molecules induced by stress: MICA/B and ULBP (5). Beside, Vδ3+ T cells are enriched in the liver and the intestine (17, 18). They can express CD4 or CD8 though the majority are double negative (CD4-CD8-). Vδ3+ T cells also express CD56, CD161, CD28, HLA-DR, and NKG2D, and some of them recognize CD1d and can exert cytotoxicity on CD1d+ target cells similar to Vδ1+ T cells (19). In humans and non-human primates, γδ T cells bearing the Vδ2 chain are the main subset present in peripheral blood and this δ chain is generally associated to the Vγ9. During infection, Vδ2Vγ9 T cells can be recruited to peripheral tissues where they contribute to the eradication of local infection (20).

**Figure 1:**
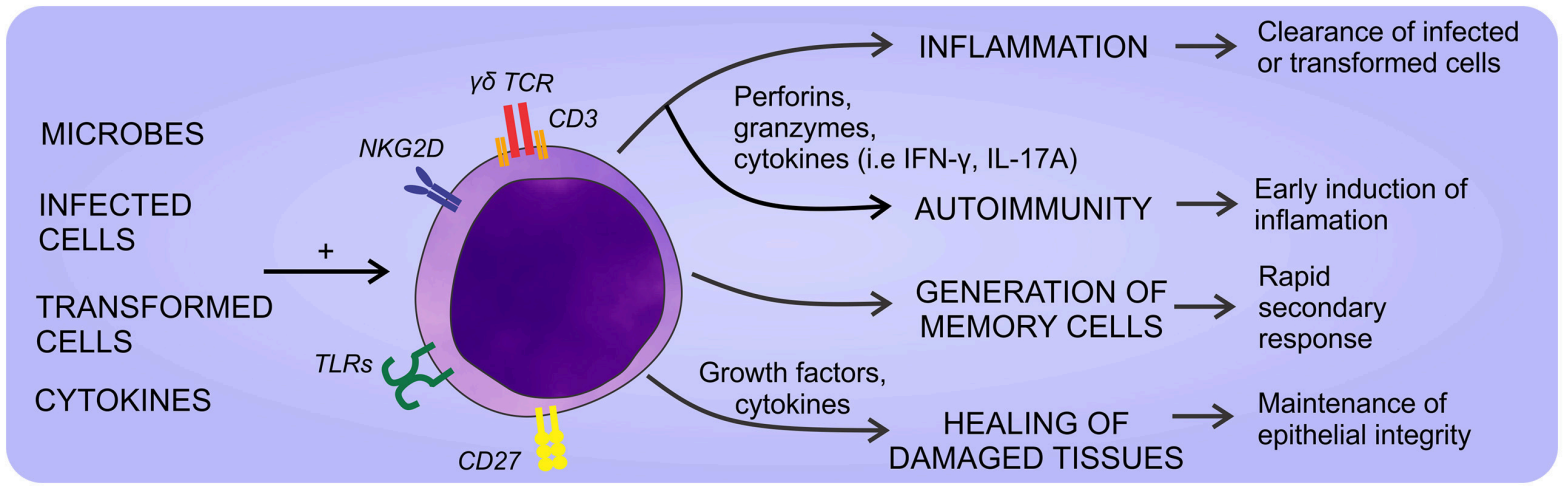
General aspects of γδ T cell physiology. γδ T cells can sense a wide array of self- and non-self-stimuli that promote different responses. Thus, they can eliminate infected or transformed cells, heal damaged tissues, and eventually promote the development of autoimmune diseases if their response is exacerbated.

Like αβ T lymphocytes, the activation of γδ T cells through the TCR requires the participation of accessory molecules. CD27 and NKG2D have been identified as co-effectors of the TCR activation (21, 22), but there is no clear consensus about the accessory molecules involved. Strikingly, in the past few years, it has been described the participation of CD277 [Butyrophilin(BTN)3A1] as a phosphoantigen presenting molecule specific for Vγ9Vδ2 TCR. According, phosphoantigen recognition is not restricted to the presentation in MHC molecules and it is independent of professional antigen presenting cells, but requires cellular contact and non-polymorphic presenting molecules (23). The main above mentioned functions reported for γδ T cells are summarized in Figure 1.

## γδ T cells in infection

γδ T cells are key effectors in the immune response against microorganisms. In many microbial infections, the number of γδ T cells increases locally and/or systemically after a few days post-infection, being able to reach a 50% of the total circulating T cells (24). A hallmark of γδ T cells is that they can recognize a broad spectrum of endogenous and exogenous antigens widespread in nature, i.e., bacteria, protozoa, and infected or transformed host cells (4). To recognize these ligands, γδ T cells employ the TCRs and receptors such as TLRs, NOTCH, NKG2D (1, 24). The rapid effector responses elicited in infectious processes are similar to those generated by innate immune cells, a property related to their ability to be activated without an antigenic priming (5). γδ T cells can directly kill infected cells by releasing the content of cytotoxic granules and bacteriostatic or lytic molecules such as granulysin and defensins (7, 25). Furthermore, they have an indirect action on the elimination of microbes by producing cytokines that promote inflammation and by inducing the antibacterial functions of immune and epithelial cells (26). As we previously mentioned, γδ T cells can differentiate into different effector profiles depending on the pathophysiological context. They can produce IFN-γ and TNF-α in response to intracellular pathogens, IL-4, IL-5, IL-13 during parasite immune responses, and IL-17 in defense against extracellular bacteria and fungi (27). Accordingly mice lacking this cell subset (TCRδ KO mice) are more susceptible to suffer infections by bacteria (*Nocardia* spp., *Klebsiella* spp., *Listeria* spp., *Escherichia coli, Salmonella* spp., *Mycobacterium* spp., and *Pseudomonas* spp.) and parasites (*Plasmodium spp*.), demonstrating a critical role of IL-17-producing γδ T cells in these processes (28). The basis of the effector function of this T cell subset is controlling neutrophil recruitment in inflamed tissues. Interestingly, at sites of inflammation, neutrophils not only exert their microbicidal activity but also regulate (inhibit or stimulate) γδ T cell functions, as it has been extensively demonstrated (29–31).

**Figure 2:**
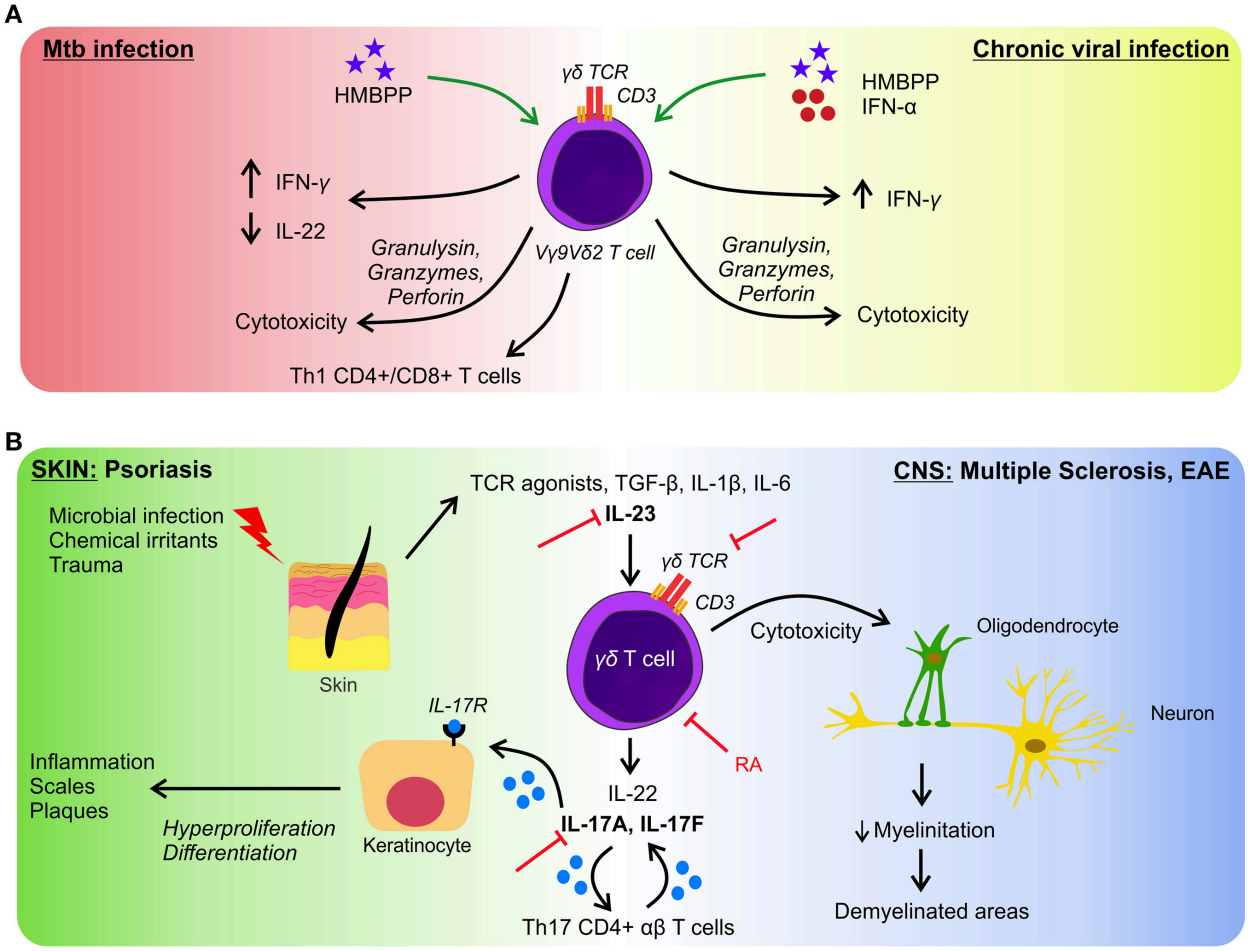
γδ T cells in infection and autoimmunity. **(A)** In response to Mtb infection, γδ T cells produce inflammatory cytokines and exert cytotoxicity on infected cells (left side), similar effector functions are performed in response to several viruses (right side). But in chronic infections γδ T cells are less effective to control microbes. Green arrows represent the proposed approaches to boost the activation of γδ T lymphocytes. **(B)** γδ T cells participate in the initiation and development of autoimmune diseases. As examples we represent pathologies in skin (left side) and in CNS (right side) both having in common an axis governed by the activation of γδ T cells and by the production of IL-17 and IL-22. Figure shows different targets to block autoimmunity manifestations (red lines). RA, retinoic acid.

Microbial recognition by Vγ9Vδ2 T cells involves phosphoantigens which are non-peptidic low molecular weight antigens with phosphate moieties, which are not only produced by prokaryotic but also eukaryotic cells. However, microbes' phosphoantigens are extremely potent activators of Vγ9Vδ2 T cells in contrast to endogenous phosphoantigens i.e., isopentenyl pyrophosphate (IPP) which is 10,000-folds less effective to induce cellular activation (32, 33). Noteworthy, eukaryotic cells under increased metabolic activity, can augment the production of IPP, i.e., tumor cells, and consequently activate γδ T cells efficiently (18). The phosphoantigen (E)-4-hydroxy-3-methyl-but-2-enyl pyrophosphate (HMBPP), an intermediate of the non-mevalonate pathway, generated by many bacteria, among them *Mycobacterium tuberculosis* (Mtb), *Mycobacterium bovis, Listeria monocytogenes, E. coli, Salmonella typhimurium*, and certain parasites such as *Plasmodium falciparum* and *Toxoplasma gondii* is an extremely potent activator of Vγ9Vδ2 T cells (33, 34). Thanks to the presence of this metabolite, Vγ9Vδ2 T cells can be activated, proliferate and produce Th1-cytokines (IFN-γ and TNF-α) (29), thus mounting a rapid response against the microbes. Moreover, during Mtb or *L. monocytogenes* infections they produce IL-17 which prompts the recruitment of neutrophil and their immune response (35). In acute infections by Mtb and HMBPP-producing microbes, this cell subset expand and in re-infections they mount a secondary memory-like response (36). Furthermore, the production of IFN-γ by stimulated-Vγ9Vδ2 T cells may contribute to the immune response against Mtb as well as to control tuberculosis lesions since they are present in lung granuloma (37). Vγ9Vδ2 T cells also limit the development of intracellular Mtb by the action of perforins, granzymes, and granulysin (20). Additionally, they can promote airway CD8+ and Th1 CD4+ responses of conventional T cells specific for Mtb, through the production of IL-12 in response to phosphoantigen activation (20). In a non-human primate model of Mtb infection, *ex vivo* activation of Vγ9Vδ2 T cells by exogenous HMBPP up-regulates their IFN-γ production. This treatment promotes the inhibition of IL-22 production, which is associated with severe lesions (38). These results might be helpful to develop novel therapeutic strategies to control Mtb infection and persistence and to induce the activation of immune cells by IFN-γ in order to eliminate intracellular Mtb (Figure 2A).

In patients with viral infections, Vδ3+ T cells are enriched. In hepatitis C virus (HCV) infections, it has been observed the expansion of several Vδ3+ T cell clones in peripheral blood (39). In the liver, these cells can mount a response against virus-infected hepatocytes and non-infected host cells, suggesting that they may contribute to the hepatic damage (40). Additionally, there is a higher frequency of IFN-γ-producing Vδ1+ cells, which correlates with disease evolution (41). During the immune response against viral infections, the recognition of non-classical MHC molecules by Vδ2- T cells is determinant but also participate Vγ9Vδ2 T cells. It has been demonstrated that activated Vγ9Vδ2 T cells can inhibit sub-genomic HCV replication by the production of IFN-γ (41, 42). In the same way, patients suffering chronic hepatitis B virus (HBV) infection, have a reduction in the circulating Vδ2+ T cells, in the production of IFN-γ and in the cytotoxicity mediated by γδ T cells. These events correlate with the persistence of HBV infection (43). Noteworthy, in mouse models of infection by West Nile virus and herpes simplex virus type 2, it has been shown that γδ T cells play a critical role in the generation of conventional CD8+ and CD4+ memory T cells, respectively (44, 45). Importantly, γδ T cells also participate in anti-viral response early in life. It has been reported that they can mount a functional immune response to cytomegalovirus infection during development in uterus, pointing out the key role of γδ T cells in fetal life (46).

Furthermore, γδ T cells participate in antifungal immunity. It has been reported that Vδ1+ T cells can selectively respond to *Candida albicans*, by producing high levels of IL-17 (47).

Given the beneficial role of Vγ9Vδ2 T cells in the clearance of microbes, the *in vivo* effect of T cell activation by phosphoantigens administered exogenously was tested in primates (48). In an infection model induced by *Yersinia pestis*, phosphoantigen treatment provoked faster pathogen clearance and restoration of inflamed tissues (49). Moreover, during chronic viral infections, where Vγ9Vδ2 T cells are decreased and their functions are impaired (50, 51), it has been proposed the administration of phosphoantigens to help restore the γδ T cell functions. Furthermore, in a non-human primate model, it was reported that after administration of HMBPP, the plasma levels of IFN-γ increased, and this effect was even higher when administered with IFN-α (52), showing a new approach to boost Vγ9Vδ2 T cell response in viral infection (Figure 2A). In this work authors also explored, *in vitro*, the effect of this combined therapy in HCV-infected patients obtaining the same results (52).

## γδ T cells in autoimmunity

It is well established that IL-17A plays a crucial role in the development and progression of autoimmune diseases (53, 54). Even though the main source of IL-17A is the Th17 CD4+ αβ T cell population, in the onset of autoimmune pathologies, innate immune cells, especially those belonging to the γδ T cell subset, also contribute to the production of IL-17A (55). Human IL-17A-producing γδ T cells are generated in the periphery and can be recruited to inflamed tissues where they accumulate (56, 57). This process takes place more rapidly compared to the activation of conventional T lymphocytes. In fact, γδ T cells can be activated in the absence of a cognate TCR ligand which allows them to be powerful early inducers of inflammation in autoimmune diseases. As demonstrated *in vitro*, several molecules are involved in the differentiation into the Th17 cytokine-profile, among them: TCR agonists, IL-1β, IL-6, IL-23, and TGF-β (57, 58). Interestingly, in patients with autoimmune liver disease such as autoimmune hepatitis, primary sclerosing cholangitis, or primary biliary cirrhosis, there is a significantly increase of γδ T cells (Vδ1+, Vδ2+, and Vδ3+) in peripheral blood and liver, supporting the participation of this subset in autoimmunity (18).

In the next paragraphs, we summarize the published data describing the role of γδ T cells in psoriasis and multiple sclerosis as two examples of autoimmune diseases where the role of γδ T cells has been extensively studied.

### Autoimmunity in skin

In steady-state conditions, in the skin and the intestine, γδ T cells are abundant and in conjunction with other immune cells, they act as sentinels and support the integrity of the epithelial barriers (59, 60). In human skin, local γδ T cells display an oligoclonal repertoire governed by the expression of Vδ1 chain (61). A well-characterized inflammatory condition in the skin is psoriasis. It is an autoimmune disease which can be triggered by microbial infections, chemical irritants or trauma. Once the pathological process starts, the innate and adaptive immune system activate and result in the hyperproliferation and the aberrant differentiation of keratinocytes, a key step in the pathophysiology of psoriasis. There is also an increase in the levels of IFN-γ and IL-23, which cause an immune-mediated dermatosis with skin lesions (62). In *in vivo* murine models of psoriasis induced by Imiquimod (TLR7/8 agonist) (63), γδ T cells were found to be necessary and sufficient to trigger skin lesions such as plaque formation, with a critical role of the axis IL-23/IL-17/IL-22. In fact, dermal γδ T cells easily proliferate and produce IL-17A, IL-17F, and IL-22 in response to IL-1β and IL-23 stimulation (63). Remarkably, γδ T cells have been proposed to initiate and precede the participation of conventional Th17 cells in psoriasis (64). In accordance, the genetic deletion of IL-17A, IL-17F, and IL-22 has shown to protect mice from Imiquimod-induced inflammation (65). Similarly, human dermal IL-17-producing γδ T cells appear to play a pathogenic role in psoriasis, as supported by evidence indicating an abundance of γδ T cells in skin biopsies from psoriasis patients, which upon stimulation with IL-23 *in vitro*, increase the IL-17 production to levels higher than αβ T cells (63). Moreover, a reduction in peripheral blood CLA+ CCR6+ Vγ9Vδ2+ T cells is observed in psoriasis patients which correlates with the severity of the pathology (66). The γδ T cells present in psoriasis skin are the Vδ1+ subset and the recently reported Vδ2+ recruited from the blood. However, this finding remain controversial because Vγ9Vδ2+ T cells are normally rare in the dermis and exhibit a low capacity to produce IL-17 (67).

Interestingly, there is different preclinical and clinical data concerning the therapeutic strategies to treat psoriasis. Based on the components involved in the onset and progression of the disease, several molecular target has been studied. Remarkably, drugs that target IL-17 and IL-23 have shown notable efficacy (68), and some of them have been licensed to treat moderate and severe psoriasis (68, 69). Given the active role of γδ T cells in the development of autoimmunity, it is possible to speculate that the mentioned immunotherapies could act not only on conventional Th17 T cells but also on γδ T lymphocytes because they express IL-23R and produce substantial quantities of IL-17A and IL-22 (Figure 2B).

### Autoimmunity in the central nervous system

Even if the role of γδ T cells in multiple sclerosis (MS) has not been completely elucidated, numerous studies have found that γδ T cells are associated with this pathology. It has been reported that γδ T cells are cytotoxic against oligodendrocytes, which participate in the myelinization of neurons, therefore, γδ T cells are implicated in the pathogenesis of MS and in its murine model, the experimental autoimmune encephalomyelitis (EAE) (70, 71). Noteworthy, it has been shown in patients suffering MS that γδ T cells accumulate in plaques and in chronically demyelinated areas of the central nervous system (CNS); and that IL-17A-producing γδ T cells increase in cerebrospinal fluids and in brain lesions (72, 73). As expected, in peripheral blood and CNS IL-17 is elevated (74). Recently it has been reported that circulating Vδ2+ T cells are decreased in MS, and it was found a negative correlation between the percentages of Vδ2+Vγ9+ T cells and the disease severity (75). These findings led to suggest that the decrease in Vδ2+ T cells impaired an effective control of auto-reactive αβ T lymphocytes (75). Additionally, in the CNS of mice with EAE, different subsets of γδ T cells were identified, among them the more abundant are: Vγ1, Vγ4, Vγ5, and Vγ6. These T cells infiltrate the brain and spinal cord in the early phases of EAE (10, 76–78). Interestingly, these subsets display different cytokine profiles, being the Vγ4+ cells, the most abundant, and the ones that produce high levels of IL-17 (76, 79). Thus, γδ T cells could be the initiators of the inflammation and the inductors of Th17 cells by producing IL-17 and IL-21 in the early phases of EAE, causing the amplification of Th17 responses (10). Of note, γδ T cells also play a beneficial role during EAE, as mediators in the resolution of the inflammation. The subset involved in the tissue repairing phase is suggested to be Vγ1, which could enhance the effector function of the regulatory T cells recruited (76), inhibit the differentiation to Th17 profile by the production of IFN-γ (80), and trigger apoptosis of pathogenic CD4+ T cells through the Fas-FasL pathway.

*In vivo* experimental data support that γδ T cells have a deleterious role in MS, i.e., in the relapsing-remitting EAE model, treating mice with TCRδ depleting antibodies immediately before the onset or during the chronic phase of the disease produces a reduction of the disease (81); and in knockout mice for IL-1RI the severity of the EAE is very decreased, demonstrating the participation of IL-1β in the induction of IL-17-producing T cells (82). Interestingly, another molecule that could modulate γδ T cells in EAE is retinoic acid. Raverdeau and co-workers demonstrated that retinoic acid treatment suppressed the production of IL-17A by murine γδ T cells *in vivo* and they also observed a reduction in the number of γδ T cells infiltrating the CNS (83). Altogether, these data show the role of new molecules that could be used to design immunotherapeutic strategies, providing new alternatives to treat autoimmunity (Figure 2B).

## Concluding remarks

In the last few years, immunotherapies based on γδ T cells have gained a great interest, supported by the anti-microbial and anti-tumor capabilities of these cells. Interestingly, these cells could be suppressed when their response is exacerbated such as in autoimmunity or some infectious conditions or could be stimulated when their response is not optimal i.e., in chronic infections. Moreover, γδ T cells can be manipulated *ex vivo* or *in vivo* to achieve an efficient immune response against infected or transformed cells. Nevertheless, further studies are necessary to address the most beneficial therapeutic approaches to modulate the self and non-self-immune response mediated by γδ T cells.

## Author contributions

CS designed and performed figures and revised the manuscript. CJ wrote the manuscript.

### Conflict of interest statement

The authors declare that the research was conducted in the absence of any commercial or financial relationships that could be construed as a potential conflict of interest.
